# Tumour-infiltrating lymphocytes are correlated with higher expression levels of PD-1 and PD-L1 in early breast cancer

**DOI:** 10.1136/esmoopen-2016-000150

**Published:** 2017-05-02

**Authors:** Atsuko Kitano, Makiko Ono, Masayuki Yoshida, Emi Noguchi, Akihiko Shimomura, Tatsunori Shimoi, Makoto Kodaira, Mayu Yunokawa, Kan Yonemori, Chikako Shimizu, Takayuki Kinoshita, Yasuhiro Fujiwara, Hitoshi Tsuda, Kenji Tamura

**Affiliations:** 1 Department of Breast and Medical Oncology, National Cancer Center Hospital, Tokyo, Japan; 2 Department of Medical Oncology, Cancer Institute Hospital, Tokyo, Japan; 3 Division of Pathology and Clinical Laboratories, National Cancer Center Hospital, Tokyo, Japan; 4 Department of Breast Surgery, National Cancer Center Hospital, Tokyo, Japan; 5 Department of Basic Pathology, National Defense Medical College, Saitama, Japan

**Keywords:** breast cancer, tumor-infiltrating lymphocytes, PD-L1, PD-1

## Abstract

**Background:**

The presence of tumour-infiltrating lymphocytes (TILs) is a favourable prognostic factor in patients with early breast cancer. Programmed cell death-1 (PD-1) and its ligand PD-L1 are associated with a variety of adverse features. The purpose of this study was to clarify the relationships between TILs, PD-1 and PD-L1 as well as their prognostic implications in early breast cancer.

**Methods:**

We investigated 180 patients with breast cancer who received neoadjuvant chemotherapy and underwent subsequent surgery for stage II–III invasive breast carcinoma between 1999 and 2007. TIL expression was classified as low or high using a previously reported scoring model. PD-1 and PD-L1 expression levels were determined by immunohistochemistry. The correlation between PD-1 expression in TILs and PD-L1 expression in cancer cells was also investigated.

**Results:**

Higher tumour grade was significantly correlated with PD-L1 expression in tumours (p<0.0001). PD-1 and PD-L1 expression levels were associated with tumour subtype and were highest in triple-negative tumours (p<0.0001). Furthermore, expression of each of PD-1 and PD-L1 was significantly correlated with higher TIL expression and pathological complete response (pCR) (p<0.0001). PD-L1 expression in cancer cells was significantly correlated with PD-1 expression in TILs (p=0.03). The correlations between pCR and expression of each of PD-L1 and PD-1 were not significant.

**Conclusion:**

Expression of PD-L1 and PD-1 in early breast cancer is associated with higher TIL scores and pCR; conversely, expression of these proteins correlates with poor prognostic clinicopathological factors such as tumour grade and subtype. TILs, PD-1 and PD-L1 can potentially predict the response to treatment.

**Key questions**

**What is already known about this subject?**

- The presence of tumour-infiltrating lymphocytes (TILs) is a prognostic factor in patients with early breast cancer.
- Programmed cell death-1 (PD-1) and its ligand PD-L1 are associated with a variety of adverse features.

**What does this study add?**

- Expression of PD-L1 and PD-1 in early breast cancer is associated with higher TIL expression and pathological complete response (pCR).
- PD-L1 expression in cancer cells was significantly correlated with PD-1 expression in TILs.

**How might this impact on clinical practice?**

- As immune-targeting therapies based on PD-1/PD-L1 blockade are being developed, understanding the roles of TILs, PD-1 and PD-L1 in breast cancer is critical. Our data show that PD-L1 and PD-1 are associated with poor clinicopathological features and (paradoxically) are indicators of good prognosis, higher TIL expression and pCR.

## Introduction

Blocking the immune checkpoint receptor ‘programmed cell death-1’ (PD-1), and its ligands PD-L1 and PD-L2, is one of the most promising strategies in cancer immunotherapy.^1–4^ PD-L1 and PD-L2 are expressed by antigen-presenting cells such as macrophages or B-cells. After binding to its ligands, PD-1 attenuates lymphocyte activation and promotes T-regulatory cell development and function, which in turn downregulates the immune response.^2–5^

Tumour-infiltrating lymphocytes (TILs) comprise various types of lymphocytes; their activities are regulated by complex immune system activator and inhibitor pathways.^5 6^ TILs have long been associated with clinical treatment response in various solid tumours.^7^ TIL expression in the breast cancer tumour microenvironment has long been recognised as a favourable prognostic factor,^8 9^ especially in tumours with highly proliferative characteristics such those that are triple-negative breast cancer (TNBC) or are human epidermal growth factor receptor 2 (HER2) positive.^10–12^ Moreover, higher TIL expression is a predictive marker of pathological complete response (pCR) following neoadjuvant chemotherapy in TNBC, as previously reported by our group and others.^13–16^

Recent studies have revealed that PD-1 and PD-L1 expression levels in breast tumours are associated with adverse clinicopathological features.^17 18^ Based on these findings, several clinical trials targeting PD-1 and PD-L1 were recently conducted.^19^ Among patients who received neoadjuvant chemotherapy, PD-L1 expression in the epithelium was a significant predictor of pCR.^20^ Sabatier *et al* also reported that high PD-1 and PD-L1 expression was associated with better overall survival and higher pCR rates in TNBC.^21^

There has been lack of studies aimed at clarifying the relationship between PD-1, PD-L1 and TILs in patients with similar-stage breast cancer. Furthermore, the associations between the expression of these proteins and clinical outcomes in breast cancer subtypes other than TNBC have not been investigated. Therefore, we performed this study to identify clinicopathological factors associated with PD-L1 expression in tumour cells and PD-1 expression in TILs in early breast cancer and to reveal the association between clinical outcomes and PD-L1 or PD-1 expression.

## Material and methods

### Patients and tissue samples

Our patient cohort was the same as that employed in our previous study on the association between TILs and neoadjuvant chemotherapy response.^13^ One hundred and eighty patients who received neoadjuvant chemotherapy and subsequent surgery for stage II–III invasive breast cancer between 1999 and 2007 with available surgical core needle biopsy (CNB) specimens were analysed. Clinical characteristics of all patients were obtained from their medical charts. All patients received anthracycline and taxane-based neoadjuvant chemotherapy using sequential or concurrent regimens (adriamycin 60 mg/m^2^ plus cyclophosphamide 600 mg/m^2^ (AC) or cyclophosphamide 600 mg/m^2^ plus epirubicin 100 mg/m^2^/5-fluorouracil 600 mg/m^2^ (FEC), followed by weekly paclitaxel 80 mg/m^2^ or triweekly docetaxel 75 mg/m^2^, or Adriamycin 50 mg plus docetaxel 60 mg/m^2^) an anthracycline-based regimen alone (AC or FEC) or a taxane-based regimen (weekly paclitaxel or triweekly docetaxel) alone. Trastuzumab was not used for 42 patients with HER2 because it was not approved for neoadjuvant chemotherapy in Japan at that time. The median follow-up time was 115 months (range: 4–202 months). All specimen were formalin fixed and paraffin embedded, and 4 µm thick sections were prepared for H&E staining. The immunohistochemistry (IHC) samples were reviewed by two observers including an experienced pathologist. The definition of pCR was ypT0/is ypN0/+ according to the National Adjuvant Breast and Bowel Project protocol.

The present study was approved by the Institutional Review Board of the National Cancer Center Hospital.

### Histopathological evaluation

Histopathological assessment of predictive factors was performed for the CNB specimens. Histopathological parameters included histological grade,^22^ histological type,^23^ presence of TILs, PD-1 expression in TILs and PD-L1 expression in tumour cells. TILs were evaluated using a previously reported TIL scoring model.^13^ Briefly, the TIL score is the sum of proportional scores (areas infiltrated by lymphocytes) and intensity scores (intensity of lymphatic infiltration) for each tumour. TIL scores of 3–5 were classified as high, whereas scores of 0–2 were classified as low.

## Immunohistochemistry

**Figure 1:**
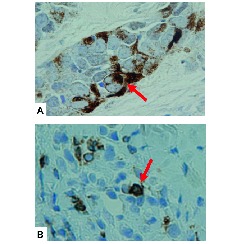
Immunohistochemical features of PD-L1 and PD-1 expression. (A) PD-L1 expression in tumour cells; the arrow indicates PD-L1 staining. (B) PD-1 expression on tumour-infiltrating lymphocytes; the arrow indicates PD-1 staining. PD-1, programmed cell death-1; PD-L1, programmed cell death-ligand 1.

IHC of the CNB specimens was evaluated using the following primary antibodies: antioestrogen receptor (ER) (clone 1D5; Dako), antiprogesterone receptor (PgR) (clone PgR636; Dako), anti-HER2 (polyclonal; HercepTest II; Dako), antihuman PD-1 monoclonal antibody (clone NAT105, 1:100 dilution; Abcam, Cambridge, UK) and antihuman PD-L1 polyclonal antibody (#4059, 1:200 dilution; ProScience, Poway, California, USA). Antigen retrieval for PD-1 was performed in TRS buffer (Dako) at 121°C for 10 min; no antigen retrieval was performed for PD-L1. The peroxidase-based Envision System (Dako) was used for secondary detection of the antibody; 3, 3’-diaminobenzidine tetrahydrochloride was used for the immunoperoxidase reaction, and the samples were counterstained with haematoxylin. Tonsil tissues were used as positive controls, while similarly treated samples absent the primary antibody were used as secondary controls. ER and PgR were judged as positive if the Allred score was ≥3 and as negative if the score was ≤2.^24^ HER2 overexpression was judged according to the American Society of Clinical Oncology/College of American Pathologists guidelines of 2007.^25^ TNBC tumours were, by definition, negative for ER, PgR and HER2. The HR+/HER2− subtype was defined as positive for ER or PgR and negative for HER2, while the HR−/HER2+ subtype was defined as negative for ER and PgR but positive for HER2. PD-1 expression in TILs and PD-L1 expression in tumours were considered positive if any positively stained cells were present (figure 1).

### Statistical analysis

Association between clinicopathological characteristics and expression of PD-1 and/or PD-L1 were evaluated using the χ^2^ test or Fisher’s exact test. Correlations between PD-1 expression in TILs and PD-L1 expression in tumours were evaluated using the χ^2^ test. Univariate and multivariate binary logistic regression analyses were performed to identify independent predictive factors of pCR. Survival curves were constructed using the Kaplan-Meier method, and statistical differences between survival curves were calculated by using the log-rank test. Statistical significance was set at p<0.05 for all the analyses, which were performed using the JMP 1.2 software, V.12.

## Results

### Patient characteristics

Table 1 shows the characteristics of the 180 patients from whom evaluable tumours were obtained using CNB. Ninety-two tumours were TNBC, 42 were HR−/HER2+ and 46 were HR+/HER2−. Patients with TNBC and HR−/HER2+ tumour subtypes had significantly higher tumour grades (p<0.001). Ninety-eight tumours (54%) had high TIL expression; high TIL-score tumours were significantly more prevalent among TNBC and HR−/HER2+ subtypes, as we reported previously.^13^ PD-1 expression was detected in TILs of 40 tumours (22%), whereas PD-L1 expression was detected in 62 tumours (34%). The PD-L1 expression rate was highest in TNBC tumours, followed by HR−/HER2+ and HR+/HER2− subtypes (p=0.004). p53 expression and the apoptosis score were higher in TNBC and HR−/HER2+ subtypes; the correlation between these factors and the three tumour subtypes was significant (p<0.001 for both factors).^13^

**Table 1:** Patient characteristics

		All (n=180)	TNBC (n=92)	HR−/HER2+ (n=42)	HR+/HER2− (n=46)	p
Age: median (range)		54 (23–76)	52 (23–76)	55 (31–71)	55 (31–71)	0.36
		Patients, n (%)				
T	1 2 3 4	2 (1) 91 (51) 54 (30) 33 (18)	2 (2) 48 (53) 27 (29) 15 (16)	0 (0) 17 (41) 16 (38) 9 (21)	0 (0) 26 (56) 11 (24) 9 (20)	0.37
N	0 1 2 3	93 (52) 67 (37) 16 (9) 4 (2)	45 (49) 35 (38) 10 (11) 2 (2)	24 (57) 14 (33) 3 (7) 1 (3)	24 (52) 18 (39) 3 (7) 1 (2)	0.96
Stage	II III	109 (61) 71 (39)	56 (61) 36 (39)	25 (60) 17 (40)	28 (61) 18 (39)	0.99
Grade	1 2 3	5 (3) 53 (29) 122 (68)	1 (1) 9 (10) 82 (89)	0 (0) 8 (19) 34 (81)	4 (9) 36 (78) 6 (3)	<0.001
TIL	Low High	82 (46) 98 (54)	25 (27) 67 (73)	19 (45) 23 (55)	38 (83) 8 (17)	0.002
PD-1 expression in TIL	Positive Negative Unknown	40 (22) 124 (69) 16 (9)	26 (28) 56 (61) 10 (11)	8 (19) 32 (76) 2 (5)	6 (13) 36 (78) 4 (9)	0.07
PD-L1 expression in tumour cells	Positive Negative Unknown	62 (34) 104 (58) 14 (8)	40 (43) 41 (45) 11 (12)	13 (31) 28 (67) 1 (2)	9 (20) 35 (76) 2 (4)	0.004
pCR	Positive Negative	41 (23) 139 (77)	29 (32) 63 (68)	9 (21) 33 (79)	3 (7) 43 (93)	0.004
p53	Positive Negative	96 (53) 84 (47)	58 (63) 34 (37)	26 (62) 16 (38)	12 (26) 34 (74)	<0.001
Apoptosis	0 1 2	59 (33) 81 (45) 40 (22)	22 (24) 51 (55) 19 (21)	8 (19) 14 (33) 20 (48)	29 (63) 16 (35) 1 (2)	<0.001

HER2, human epidermal growth factor receptor 2; HR, hormone receptors; pCR, pathological complete response; PD-1, programmed cell death-1; PD-L1,  programmed cell death-ligand 1; T, tumour; TIL, tumour-infiltrating tumour cells; TNBC, triple-negative breast cancer; N, node.

### Correlation between clinicopathological factors and PD-L1 expression in tumour cells or PD-1 expression in TILs

The correlations between clinicopathological factors and PD-L1 expression in tumour cells or PD-1 expression in TILs are shown in table 2. PD-L1 expression was evaluated in 166 tumours, while PD-1 expression in TILs was evaluated in 164 tumours among the 180 screened samples. Higher tumour grade was significantly correlated with PD-L1 expression in tumours (p<0.0001). The tumour subtype also correlated with PD-L1 expression in tumours, particularly TNBC (p<0.0001). PD-L1 expression in tumour cells was detected in all patients who achieved pCR; moreover, there was a significant correlation between achievement of pCR and PD-L1 expression in tumours (p<0.0001). Higher TIL scores and p53 expression were significantly correlated with PD-L1 expression in tumour cells (p<0.0001 and p=0.0074, respectively). Clinicopathological factors that were statistically correlated with PD-L1 expression in tumour cells likewise correlated with PD-1 expression in TILs; these included tumour grade, tumour subtype, pCR, higher TIL score and p53 expression.

**Table 2:** Correlation between clinicopathological factors and PD-L1 expression in tumour cells or PD-1 expression in TILs

		No. of patients (%)		p	No. of patients (%)		p
		PD-L1 expression (n=166)			PD-1 expression (n=164)		
		Positive	Negative		Positive	Negative	
Age (years)	≤50 >50	25 (43) 37 (35)	33 (57) 70 (65)	0.28	15 (27) 25 (24)	41 (57) 83 (65)	0.61
T	1/2 3/4	53 (61) 39 (49)	34 (39) 40 (51)	0.13	54 (62) 40 (52)	33 (38) 37 (48)	0.19
N	0 ≥1	47 (55) 45 (56)	39 (45) 35 (44)	0.84	49 (56) 45 (58)	38 (44) 32 (42)	0.78
Stage	II III	40 (40) 22 (34)	61 (60) 42 (66)	0.18	26 (25) 14 (23)	76 (75) 48 (77)	0.21
Grade	1 or 2 3	15 (28) 47 (42)	39 (72) 64 (58)	<0.0001	9 (17) 31 (28)	43 (83) 81 (72)	<0.0001
Subtype	TNBC HR−/HER2+ HR+/HER2−	40 (49) 13 (33) 9 (21)	41 (51) 27 (67) 35 (79)	<0.0001	26 (32) 8 (20) 6 (14)	56 (51) 32 (67) 36 (79)	<0.0001
pCR	Yes No	33 (100) 59 (44)	0 (0) 74 (56)	<0.0001	34 (100) 60 (46)	0 (0) 70 (54)	<0.0001
TIL	High Low	66 (76) 26 (33)	21 (24) 53 (67)	<0.0001	67 (78) 27 (35)	19 (22) 51 (65)	<0.0001
p53	Positive Negative	58 (67) 34 (43)	29 (33) 45 (57)	0.0074	59 (70) 35 (44)	25 (30) 45 (56)	0.002
Apoptosis	Score 0, 1 Score 2	69 (54) 23 (59)	58 (46) 16 (41)	0.61	71 (56) 23 (61)	55 (44) 15 (39)	0.65

HER2, human epidermal growth factor receptor 2; HR, hormone receptors; pCR, pathological complete response; PD-1, programmed cell death-1; PD-L1,  programmed cell death-ligand 1; TIL, tumour-infiltrating tumour cells; TNBC, triple-negative breast cancer.

### Correlation between PD-L1 expression in tumour cells and PD-1 expression in TILs

One hundred and sixty-two tumours were evaluable for both PD-L1 expression in tumour cells and PD-1 expression in TILs among the 180 screened samples (table 3). PD-L1 expression in tumour cells was significantly associated with PD-1 expression in TILs (p=0.03). Among PD-L1-positive tumours, 21 (34%) also expressed PD-1. The rate of coexpression of PD-L1 in tumour cells and PD-1 expression in TILs from the same specimen was 13% (21/162).

**Table 3:** Correlation between PD-L1 expression in tumour cells and PD-1 expression in TILs

n=162	PD-1 expression in TILs		p=0.03
	Positive	Negative	Total
PD-L1 expression in tumours cells (no. of patients (%))			
Positive	21 (34)	40 (66)	61 (100)
Negative	19 (19)	82 (81)	101 (100)
Total	40	122	162

PD-1, programmed cell death-1; PD-L1,  programmed cell death-ligand 1; TIL, tumour-infiltrating lymphocyte.

### Correlation between PD-L1, PD-1 expression and TILs with pCR by tumour subtype

The correlations between pCR and each of PD-L1 expression, PD-1 expression and TIL score were evaluated by using the χ^2^ test (table 4). PD-L1 expression in tumour cells and pCR were marginally correlated in HR−/HER2+ tumours, although the relationship was not significant (p=0.08). No correlations between PD-1 expression in TILs and pCR were evident in any of the tumour subtypes. High TIL scores and pCR were significantly correlated in TNBCs, which is consistent with our previous study^13^ (p=0.05); however, no such significant differences were observed in the HR−/HER2+ or HR+/HER2− subtypes.

**Table 4:** Correlation between PD-L1 expression in tumours/PD-1 expression in TILs and TIL score with pCR by subtype

	TNBC		p*	HR−/HER2+		p*	HR+/HER2−		p*
	N (%)	% pCR rate		N (%)	% pCR rate		N (%)	% pCR rate	
PD-L1 expression									
positive negative	40 (49) 41 (51)	22.5 (9/40) 36.6 (15/41)	0.16	13 (32) 28 (68)	38.5 (5/13) 14.3 (4/28)	0.08	9 (20) 35 (80)	0 (0/9) 2 (2/35)	0.46
PD-1 expression									
positive negative	26 (32) 56 (68)	30.8 (8/26) 30.3 (17/56)	0.97	8 (20) 32 (80)	25.0 (2/8) 21.9 (7/32)	0.85	6 (14) 36 (86)	0 (0/6) 8.3 (3/36)	0.46
TIL score									
High Low	67 (73) 25 (27)	37.3 (25/67) 16.0 (4/25)	0.05	23 (55) 19 (45)	30.4 (7/23) 10.5 (2/19)	0.12	8 (17) 38 (82)	12.5 (1/8) 5.3 (2/38)	0.45

*p Values were calculated between each of the three factors and pCR by using the χ^2^ exact test.

HER2, human epidermal growth factor receptor 2; HR, hormone receptors; pCR, pathological complete response; PD-1, programmed cell death-1; PD-L1,  programmed cell death-ligand 1; TIL, tumour-infiltrating lymphocytes; TNBC, triple-negative breast cancer.

### Survival outcome according to PD-L1 expression in tumour cells and PD-1 expression in TILs

**Figure 2:**
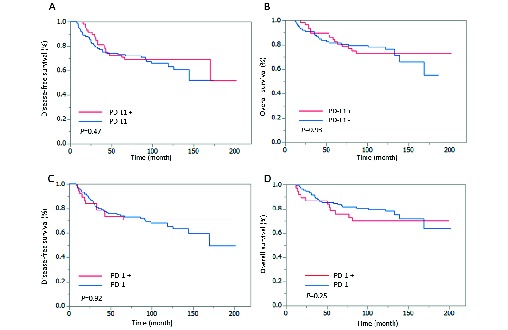
Survival outcomes according to PD-L1 or PD-1 expression. Kaplan-Meier estimates of disease-free survival and overall survival according to expression levels of PD-L1 and PD-1. Expression of PD-L1 and PD-1 did not significantly influence survival. (A) Disease-free survival according to PD-L1 expression. (B) Overall survival according to PD-L1 expression. (C) Disease-free survival according to PD-1 expression. (D) Overall survival according to PD-1 expression. PD-1, programmed cell death-1; PD-L1,  programmed cell death-ligand 1.

No significant differences in the rates of disease-free and overall survival were identified according to PD-L1 or PD-1 expression during the follow-up period (median: 115 months; range: 4–202 months) (figure 2). Furthermore, there were no statistically significant differences in survival rates according to PD-L1 and PD-1 expression by tumour subtype (data not shown).

## Discussion

Our data clarified the relationship between expression of PD-L1 in tumour cells and PD-1 in TILs. The association between certain clinicopathological factors and PD-L1 expression in breast cancer has been reported in various studies.^17 26^ A meta-analysis revealed that PD-L1 expression in breast tumours is a poor prognostic clinicopathological factor, as it is related to lymph node metastasis, poor nuclear grade and negative oestrogen receptor status.^27^ The present study similarly concluded that PD-L1 expression in tumours correlates with both poor grade and more aggressive tumour subtypes. Our findings also suggest that PD-1 expression in TILs is likewise related to poorly prognostic clinicopathological factors. Moreover, expression of both PD-L1 in tumour cells and PD-1 in TILs were related to a high TIL score and, paradoxically, a higher pCR rate.

Although PD-L1 expression in tumours is considered a diagnostic marker for anti PD-1 antibodies (such as pembrolizumab) in non-small cell lung cancer,^28^ it is still unclear whether PD-L1 expression is a prognostic marker for all immune-targeting therapies based on PD-1/PD-L1 blockade in various types of cancer. To ensure a response to PD-1/ PD-L1 checkpoint blockade, a new framework based on the presence or absence of TILs and PD-L1 expression was required.^29 30^ Four categories of tumours according to TIL and PD-L1 expression were proposed as a measure of predicting their response to immune checkpoint inhibitors: type I (PD-L1+/TIL+; adaptive immune resistance), type II (PD-L1-/TIL-; immunological ignorance), type III (PD-L1+/ TIL-; intrinsic induction) and type IV (PD-L1-/TIL+; tolerance).^31^ The relationship between PD-L1 expression and TILs in breast cancer was also reported in some retrospective clinical investigations,^20 32^ although no published data exist regarding the predictive value of PD-L1 expression and TILs for immune checkpoint inhibitor therapy to our knowledge.

Previous studies showed that PD-L1 expression correlated with that of TILs and to neoadjuvant chemotherapy response, particularly in HR− tumours and TNBCs.^20^ Our data suggest that PD-L1 expression, as well as TIL scores, have a correlative trend with pCR. We also revealed that PD-1 expression in TILs correlated with the TIL score. However, we did not reveal PD-L1 expression in tumour cells, PD-1 expression in TILs and TIL scores to be predictive markers of pCR except for TIL scores in patients with TNBC, which our group also reported previously.^13^ PD-L1 expression in tumours marginally correlated with pCR in HR−/HER2+ tumour, although not significantly.

PD-L1 and PD-1 were shown to be expressed concurrently in various tumours, including endometrial tumours (79%), malignant melanomas (58%), bladder cancer (55%), non-small cell lung carcinomas (43%), ovarian cancer (36%) and kidney cancer (33%).^33^ It was also reported that the rate of concurrent PD-L1 and PD-1 expression in breast cancers regardless of tumour subtype was 29%; however, the rate in TNBCs was significantly higher than that in non-TNBCs (45% vs 13%–17%, respectively; p=0.001).^33^ In our study, the rate of concurrent PD-L1 and PD-1 expression was 34%, which was similar to rates previously reported. Moreover, we found that PD-L1 expression in tumours was significantly associated with PD-1 expression in TILs. A previous study showed that PD-1 expression in TILs was associated with a higher rate of mutations, whereas PD-L1 expression in tumours showed the opposite association.^33^ Breast cancers with mutated *TP53* exhibit higher PD-1 in TILs compared with breast cancers that harbour other mutations (eg, *PIK3CA*) or those without mutations.^33^ In contrast, PD-L1 expression in tumour cells does not correlate with mutation status.^33^

TIL presence has been reported to be a positive prognostic factor for TNBC and HER2-positive breast cancer^8 10 22^; however, few studies have explored the role of PD-L1 and PD-1 expression in the survival of patients with breast cancer. Sabatier *et al* reported that PD-L1 mRNA upregulation was associated with better survival and response to chemotherapy in patients with TNBC.^21^ Schalper *et al* also reported that PD-L1 mRNA expression is associated with increased TILs and improved recurrence-free survival rates.^32^ Our data showed no survival differences according to PD-L1 or PD-1 expression in any of the breast cancer subtypes. One reason for this discrepancy may be owing to the use of IHC to evaluate our specimens, not mRNA expression; the discordance between mRNA and IHC-based determination of PD-L1 expression might influence our results.^20 32^ Another reason may be attributed to the fact that upregulated PD-L1 and PD-1 were not only poor prognostic markers but, paradoxically, good predictive markers of pCR. Therefore, the survival outcomes may have been obscured by these competing measures.

In summary, we showed that expression of PD-L1 and PD-1 is associated with higher TIL scores and pCR; conversely, expression of these proteins correlated with poor prognostic clinicopathological factors such as tumour grade and subtype. PD-L1 expression in tumours and PD-1 expression in TILs were significantly correlated, and 34% of all tumours concurrently expressed both proteins. However, our results did not support the notion that PD-L1 and/or PD-1 expression are predictive markers for survival, which had previously been shown in a study of mRNA PD-L1 expression levels in TNBC.^21 32^ The main strengths of our study were that both PD-L1 expression in tumours and PD-1 expression in TILs were evaluated in the same tumour specimens and that we were able to perform statistical analysis of PD-L1 and PD-1 expression in this context.

Our study has a number of limitations. One is its retrospective nature, as it was performed using archived specimens. A second limitation is that PD-L1 and PD-1 expression levels were evaluated by IHC and not transcriptionally. A third limitation is that our patients had received neoadjuvant chemotherapy using cytotoxic regimens, not immune checkpoint inhibitors. Namely, PD-L1 or PD-1 expression in this study does not reflect immune checkpoint inhibitor efficacy. Our data indicate that expression levels of PD-L1 and/or PD-1 are not sufficient predictive factors for survival; hence, alternative frameworks that include PD-L1, PD-1 and TILs combined with other microenvironmental factors are required for reliable prognostication.
